# Digital storytelling online: a case report exploring virtual design, implementation opportunities and challenges

**DOI:** 10.1186/s40900-024-00570-6

**Published:** 2024-04-09

**Authors:** Elizabeth Mansfield, Nafeesa Jalal, Rani Sanderson, Geeta Shetty, Andrea Hylton, Chelsea D’Silva

**Affiliations:** 1 Trillium Health Partners, Institute for Better Health, 2085 Hurontario Street, Mississauga, ON L5A 4G1 Canada; 2 Department of Occupational Science and Occupational Therapy, University of Toronto, 6 Queen’s Park Crescent West, Toronto, ON M5S 3H2 Canada; 3 School of Public Health, Seneca College, 13990 Dufferin St, King City, ON L7B 1B3 Canada; 4 NJ Global Consulting Inc, 533 Scott Blvd, Milton, ON L9T 0T8 Canada; 5 StoryCentre Canada, 1 Bedford Rd, Toronto, ON M5R 2B5 Canada; 6 Community Co-researchers, Mississauga and Brampton, Brampton, ON Canada; 7 19 to Zero Inc, 4702 21 Street SW, Calgary, AB T2T 5T4 Canada

**Keywords:** Digital storytelling, Participatory methods, Arts-based methods, Lessons learned

## Abstract

**Background:**

Digital storytelling is an arts-informed approach that engages short, first-person videos, typically three to five minutes in length, to communicate a personal narrative. Prior to the pandemic, digital storytelling initiatives in health services research were often conducted during face-to-face workshops scheduled over multiple days. However, throughout the COVID-19 lockdowns where social distancing requirements needed to be maintained, many digital storytelling projects were adapted to online platforms.

**Methods:**

As part of a research project aiming to explore the day surgery treatment and recovery experiences of women with breast cancer in Peel region, we decided to pivot our digital storytelling process to an online format. During the process, we observed that the online digital storytelling format had multiple opportunities and challenges to implementation.

**Results:**

This paper outlines our promising practices and lessons learned when designing and implementing an online digital storytelling project including pre-production, production and post-production considerations.

**Conclusions:**

We provide lessons learned for future teams intending to conduct an online digital storytelling project.

## Abstract (plain language summary)

Digital storytelling uses pictures, video clips and audio to create a short, first-person video, to share a person’s story. Before the pandemic, digital storytelling workshops were often held in-person over multiple days to help members of the community create their own digital stories. However, throughout COVID-19, many digital storytelling workshops were held online instead. This paper outlines our lessons learned when hosting an online digital storytelling workshop series to capture women’s experiences with breast cancer in our local community. We hope our lessons learned are helpful for other teams who are considering using online digital storytelling workshops for their own research projects.

## Background

Digital storytelling (DST), a participatory visual method, is increasingly engaged in community-based health research and knowledge translation initiatives [1]. As an arts-informed approach, digital storytelling uses short, first-person videos, typically three to five minutes in length, to communicate a personal narrative [2–4]. In contrast with testimonial videos where the camera is focused on the speaker’s face, digital stories are personal films accompanied by the storyteller’s own voice and narrative, sounds/music and a series of selected visuals to communicate lived experience and first person accounts [5]. Digital storytelling has been used in healthcare for research [2, 6, 7], education [8, 9], community advocacy [3, 10–12], and as a therapeutic intervention [13, 14]. Additionally, digital stories have been identified as a community-based participatory research method that is especially well-suited for sharing and communicating the experiences of individuals from diverse and racialized communities [15–18]. As Lenette (2019) [19] observes, this participatory approach in healthcare initiatives can support more equitable power dynamics as community members are “privileged as protagonists-and-producers of their own stories” (p. 138).

Digital storytelling offers a process-oriented but flexible participatory approach to empower and support community members as they curate their own stories in collaboration with a trained facilitator [20]. These group-based participatory methods for creating and sharing personal narratives are reported as supporting the inclusion of individual voices, lived experiences and community perspectives in research and knowledge translation initiatives [14, 21, 22]. In this report, we use the term “community co-researcher” to highlight the collaborative and participatory role that people who have lived experience and understanding of the topic bring to research and local change initiatives by sharing their personal stories and contributing to the direction of the project [23, 24]. 

Prior to the pandemic, digital storytelling initiatives in health services research were often conducted in face-to-face venues with workshops and video production schedules typically taking place for 8 h over a consecutive three-day period [14]. These in-person workshops often include a blend of instructional education and working periods where co-researchers work on their DST collaboratively with facilitators. As this in-person format was not possible during COVID-19 lockdowns, many digital storytelling projects were adapted to online platforms while maintaining the same content [14, 17, 25]. Digital storytelling group initiatives held in virtual venues have necessitated structural and procedural changes such as adaptations to workshop session length and cadence, project onboarding processes, facilitation methods, different approaches to learning technical aspects and community co-researcher support. Post-pandemic, online digital storytelling project designs continue to be a popular option due to the virtual method’s potential for inclusivity, along with the greater convenience, comfort and feasibility of participating from home [2, 26, 27]. 

Due to the rapid proliferation of virtual research initiatives during the past few years, community-based researchers are increasingly seeking guidance on promising practices for adapting qualitative methods that are traditionally in-person to online formats [26, 28, 29]. While the topic of conducting online qualitative interviews and focus groups is addressed in the literature, a more detailed, pragmatic and contextualized understanding of best practices and considerations for online digital storytelling is also required [21, 25, 30–32]. In response to a paucity of pragmatic guidelines for online participatory visual methods research, we aim to report on promising practices and lessons learned when designing and implementing an online digital storytelling project.

## Methods

### Project background and setting

The digital story initiative reported here was the second phase of a qualitative research project exploring the day surgery treatment and recovery experiences of women with breast cancer in Peel region. Located in southwest Ontario, Peel is one of Canada’s most ethnoculturally and racially diverse communities with more than half of the region’s residents identifying as racialized individuals and/or minorities [33]. During the first phase, the qualitative project team conducted a series of 17 in-depth, semi-structured interviews with women from South Asian and Black communities - the two largest racialized populations in Peel region. During individual project follow up discussions, interview participants expressed an interest in sharing their stories in an impactful format as a catalyst for community-based breast health and cancer survivorship discussions and to advocate for culturally responsive oncology services and system change. Digital storytelling resonated with participants as an engaging knowledge translation approach for sharing lived experience of breast cancer treatment and recovery with multiple stakeholder audiences. Project staff appreciated that an online digital storytelling study design could accommodate COVID 19 physical distancing requirements as well as a growing evidence base that this participatory visual method lends itself well to adaptations for virtual settings [1, 21, 25]. 

### Study design

In preparation for the project, the core qualitative project team participated in a 6-week online digital storytelling workshop series through StoryCenter, U.S. This training opportunity allowed the qualitative research staff to experience firsthand the creation and sharing of digital stories in an online setting and subsequently informed protocol development and workshop planning in the Spring of 2021. The online workshop series demonstrated the importance of extensively trained facilitators with technical skillsets to support workshop participants in using video editing software. Experienced DST facilitators also created a welcoming and collaborative environment that was key to the success of this workshop series. Following research ethics board approval for the study’s second phase, seven interviewees from Phase 1 expressed interest in joining the digital storytelling initiative as community co-researchers. The workshop included twelve people (seven community co-researchers, three staff researchers and two DST workshop facilitation experts) and allowed both large and small group work to be conducted in an online setting. Following best practices in community-based research and patient partner engagement, honoraria were provided to participants to support equitable inclusion and compensate for their expertise and time related to both workshop sessions and individualized DST work between group meetings. Table 1 describes the steps and focus of the seven workshop sessions as well as activities conducted between sessions and post-workshop knowledge translation collaboration. Project staff members curated the workshop format in collaboration with StoryCentre Canada to ensure that the content typically included in the in-person workshops was adapted to both the online format and our participatory research project needs. During the first session, all project team members developed principles of collaboration to ensure that co-researchers were involved in co-creating social norms for the group and ensuring the environment encouraged participation.

**Table 1:** Overview of DST Workshop Sessions & Activities

Activity	Description
**Pre-meeting preparation**	**Individual 1:1 telephone/Zoom discussions** − DST Project orientation/overview & activity timeline – Q & A session two to three weeks before first workshop event − Developing principles of collaboration − Review study information and document informed consent
**Session 1: Introduction to the Project**	− Project aims & introduction to digital storytelling (DST examples) − Story prompts for storyteller feedback & revision − **Assignment**: Story ideas for sharing during Story Circle 1
**Session 2**: **Story Circle 1**	− Story sharing guidelines (time allotted for story sharing, active listening, feedback) − Story Circle activity (using break-out rooms to share ideas for digital stories) − **Assignment**: Write a rough draft 300 to 500 word script in first person communicating your narrative
**Session 3**: **Story Circle 2 for script development**	− Storyboarding/visual treatment and photo/visuals discussion − Story Circle for storytellers to share draft scripts using Zoom breakout rooms − Scheduling 1:1 recording sessions with DST facilitators − **Assignment**: Finalize script and record voice-over
**Session 4**: **Selecting visuals/sound and editing**	− Intro to basics of online video recording & editing software − Best practices for creating/selecting images, sound, music − **Assignment**: Selecting images/sounds to support the narrative voiceover and begin editing rough draft of video
**Meeting 5**: **Editing and refining DSTs**	− Sharing DSTs in progress using breakout rooms for small group feedback − DSTs refined during 1 h individual editing sessions − **Assignment**: Complete DSTs for Meeting 6
**Session 6: Celebration event**	− Celebration event where DSTs are shared − Large group discussion/feedback about project and methods and next steps − Scheduling Meeting 7 to take place after four-week break − **Assignment**: During the break, reflect on if you would like to share your story and with whom
**Session 7**: **DST sharing and collaboration plans**	− Discussion about story sharing and idea generation about where to share − Group feedback about workshop strengths, challenges, and opportunities for improvement − Planning a post-workshop communication strategy for knowledge translation (KT) activities − **Assignment**: Scheduling individual informed consent discussion for sharing DSTs for specific KT activities and project debriefing discussions

· 1:1 follow up discussions with storytellers between workshop sessions (assignment support, general check-in, workshop meeting feedback, date/time reminders for workshop sessions)

• Project staff debriefs prior to and following each workshop meeting

Throughout the digital storytelling workshops, all team members (community co-researchers, staff researchers and DST workshop facilitation experts) were encouraged to share their reflections during rounding at the start of each online meeting and during co-researcher and staff debriefs. During session 7, all team members participated in a group feedback session where they shared workshop strengths, challenges and opportunities for improvements. The staff researchers took additional notes during and after workshop sessions to capture the team’s feedback and understand what worked well during the workshop and where improvements could be made. This feedback focused on project onboarding, workshop facilitation and format, and opportunities for knowledge translation and advocacy. The discussion with the project team was used to develop the lessons learned presented in this paper. The lessons learned were further reviewed and refined by the project team to ensure they captured the team’s experiences. This was especially important given that the team included co-researchers, researchers and DST facilitators who all had varying levels of experience and knowledge related to digital storytelling.

## Results

**Fig. 1:**
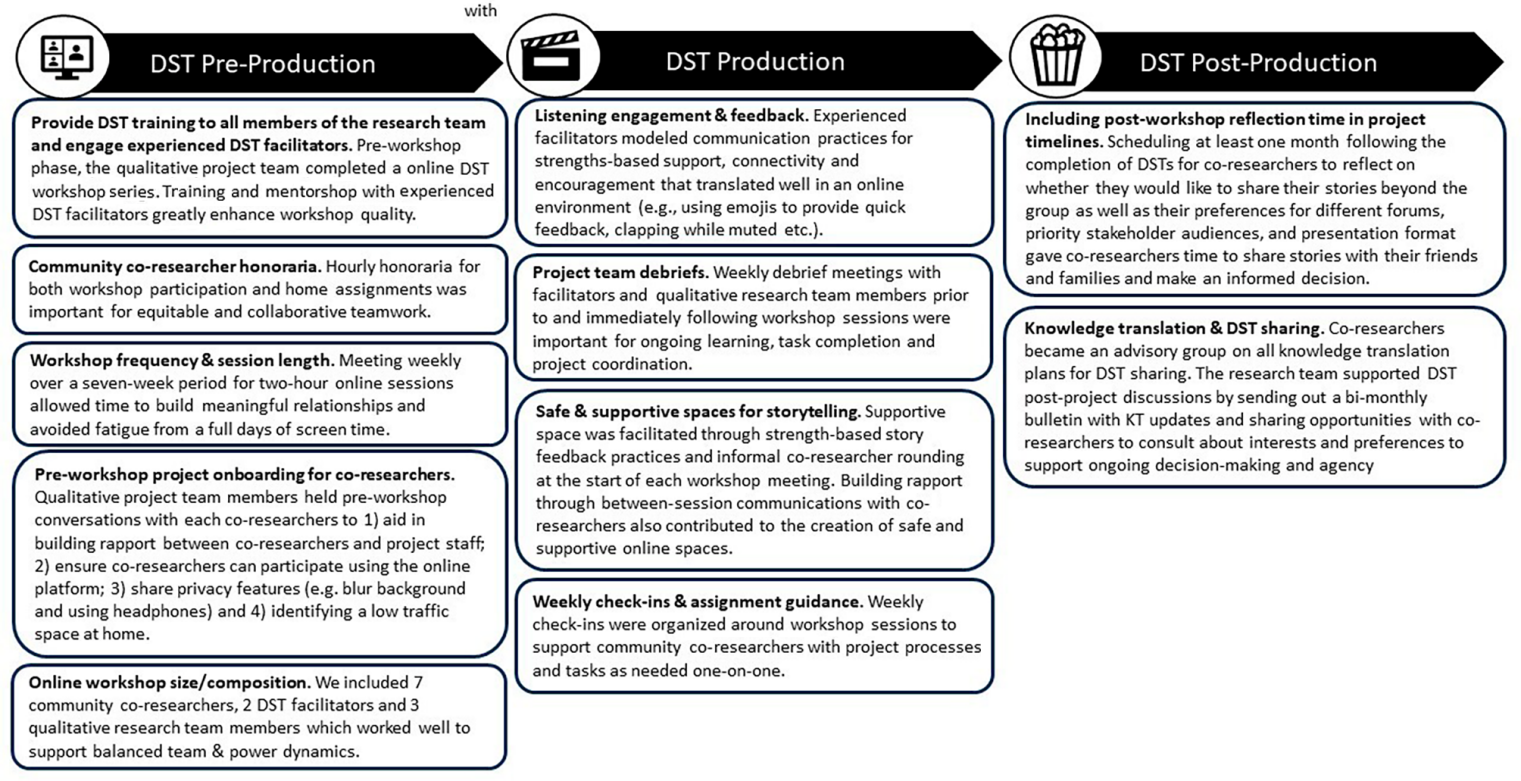
Online promising DST practices- Lessons learned and promising during three phases of digital storytelling video creation: (1) pre-production (2) production and: (3) post-production

Co-researchers developed seven individual digital stories sharing their own personal experiences throughout their breast cancer journeys. The completed digital stories can be found here [34]. A summary of the online digital storytelling insights and lessons learned from this project can be found in Fig. 1.

### Comprehensive pre-workshop staff training is required:

Providing education for the qualitative project team before developing the protocol and launching the project was very beneficial. Through participating in a virtual DST workshop series, project staff gained a nuanced appreciation of the collaborative project processes and task requirements for co-researchers creating DSTs in a virtual setting. This training highlighted both the importance of participatory methods such as active listening and experience-based feedback and how to communicate these practices in online DST workshop environments. As well, training provided firsthand experiences with DST methods and modalities that supported project onboarding discussions, working with storytellers virtually between workshop meetings and online knowledge translation approaches such as webinars and virtual presentations. Having access to pre-project training in online DST methods along with the guidance of skilled and experienced DST facilitators were factors contributing to project staff members’ ability to learn and engage with new research practices. Experienced DST facilitators at StoryCenter have all taken an initial storytelling workshop and through an apprenticeship training program, complete a 5-day facilitator-intensive-training course and shadow multiple workshop series, before co-facilitating and then leading their own workshops. Many have been facilitating for over 15 years and have a teaching background which adds to their experience and ability to support co-researchers. The DST facilitators we worked with scheduled regular debriefs/planning sessions to discuss what worked well and what we could improve upon, based on project staff and community co-researcher feedback. For example, building in social time at the start of each workshop session to share personal as well as project progress updates was a practice that supported a collaborative and strengths-based environment. During early workshop sessions, co-researchers prioritized sharing their contextualized, lived health experiences over discussions of technical topics such as video editing. As a result, we provided technical instructions during individual sessions.

### The importance of pre-workshop project onboarding conversations:

With the online format and potential for fatigue, we limited the duration of individual workshop sessions to 2 h. Given this shortened amount of time, the qualitative project team facilitated a series of individual pre-workshop discussions with community co-researchers over Zoom. During the first pre-workshop one-to-one meeting, the study information sheet and consent form for group workshop participation were reviewed and weekday evening workshop dates and time preferences recorded. Four to six weeks prior to the workshop launch, additional individual onboarding conversations took place and included the following topics: the participatory group approach, project design, time commitment, technology supports/familiarity and online workshop logistics. Co-researchers were encouraged to share any suggestions or accommodations that may be needed in order to participate in the workshops. Online workshop logistics discussions addressed included internet connectivity, computer access, and familiarity with Zoom, use of headphones for privacy along with the availability of a quiet, low traffic home space. These pre-workshop meetings also included viewing examples of digital stories to enhance an understanding of the format for participants unfamiliar with this arts-based method along with online DST production procedures. Pre-workshop communications also involved conversations about storyteller ownership, control, agency and choice to share or not share DSTs. These pre-workshop meetings were important in preparing participants for their roles as community co-researchers actively engaged in decision-making and DST planning. The conversations strengthened rapport and trust between community co-researchers and project staff to support subsequent online workshop activities, DST task completion and knowledge translation planning. Allowing sufficient time and resources for comprehensive project onboarding and Q & A discussions was critical to the success of this initiative.

### Benefits of longer workshop timelines for online settings:

Translating a three full-day workshop format, popular with in-person initiatives, to a virtual digital storytelling design, required adaptations to mitigate the risks of screen fatigue and maintaining focus in online environments. Recognizing these challenges, the breast cancer and diversity digital storytelling project was adapted to include seven weekly two-hour meetings with individual coaching time and check-ins scheduled to support work between meetings (Table 1). Individual online check-ins and coaching time acted as a proxy for informal conversation opportunities that typically take place on the fly during in-person workshops. A benefit of this seven-week period for DST workshops was the opportunity to develop a supportive, interpersonal network over time. For example, community co-researchers communicated with one another through social media messaging apps and shared information about community resources and support groups. Sharing a monthly newsletter, providing project updates and DST sharing opportunities, further supported community co-researcher connectivity. The extended timelines of an online DST project were advantageous in developing rapport and meaningful relationships among co-researchers.

### Active listening and feedback practices contributed to collaborative and engaged online workshop events:

Initially, project staff members were concerned that even a two-hour online workshop session conducted over Zoom might be too long and result in screen fatigue and less engaged participation. However, the implementation of story circle practices and active listening techniques with the guidance of expert DST facilitators helped mitigate this challenge. Workshops included dedicated segments of approximately ten minutes of uninterrupted time for each community co-researcher followed by five minutes of group feedback and discussion. DST facilitators modeled strengths-based feedback techniques, well-suited for online environments. For example, the group was encouraged to express support through physical gestures such as applause and placing hands over hearts. The facilitators also asked inidivdiuals to not check emails or phones during this time so that they were fully present for their co-researchers. Written feedback using the Zoom chat function was encouraged after, rather than during, individual storytelling segments. By modeling these respectful and non-distracting feedback techniques, the workshop facilitators encouraged a strengths-based environment for sharing lived experience. These intentional communication practices were experienced by our group as contributing to a sharing environment that minimized cross-talk and fostered deep engagement. Using online breakout rooms for small group discussion and focused feedback also supported community co-researcher collaboration. The size of our group (seven community co-researchers, three project staff members, two DST facilitators) and balanced representation of community co-researchers and project staff was optimal for both large group and breakout room activities.

### Optional between workshop meetings with community co-researchers supported ongoing project engagement:

Digital storytelling projects require the completion of workshop assignments in between meetings including developing and refining a short narrative, recording a voice-over narrative, creating/retrieving visual images (e.g. photos, artwork, graphics), selecting music and audio. DST facilitators and project staff met online/remotely with community co-researchers to support and provide guidance between meetings. The scheduling of individual and small group coaching sessions supported video narrative development and community co-researcher engagement that helped sustain project momentum. Weekly check-ins between workshop sessions that were task-driven also created an opportunity and safe space for community co-researchers to discuss how they were experiencing the project and to identify requirements for additional supports and/or resources.

### Taking time between digital story creation and knowledge translation planning:

To ensure there was time to reflect upon decisions related to the broader sharing of digital stories, the group opted for a one-month break between the final DST workshop session and knowledge translation session (Meeting 7). This break created time and space for community co-researchers to share their stories with their family and friends while reflecting upon whether they wanted to share their stories, how and where to share and with whom. During the knowledge translation session, co-researchers discussed their experiences of previewing their films with family, friends and personal networks. As family-friends were included or referenced in several of the digital stories, their endorsement was an important step for co-researchers before considering opportunities to share the stories in public forums. For broader digital storytelling and project results sharing, we agreed upon an engaged consensus decision-making approaches. Community co-researchers could opt in or decline participation to present at conferences and other knowledge translation opportunities. The community co-researchers contributed to all knowledge translation activities and played an active role in reviewing and selecting conference, education, and online sharing opportunities. The digital stories were presented as a collection at national and international virtual and in-person conferences and learning sessions with graduate students. The group continues to explore future funding opportunities, advocacy and research opportunities for co-researchers and has recently launched a project webpage (healthexperiences.ca) for accessing the DSTs, community co-researcher bios and project background context. Through bi-monthly communications over email and Zoom, community co-researchers communicated DST sharing preferences and also recommended different venues and networks for socializing their work.

### Early community and stakeholder engagement:

Protecting sufficient funds for knowledge translation and community outreach activities is also an important lesson learned. For future work, we would recommend engaging community co-researchers and community partners through the creation of a community advisory board in the early planning phases to inform all stages of digital storytelling projects, including workshop design and knowledge translation in particular. Although many healthcare organizations and community partners support the inclusion of lived experience, stakeholders are often unfamiliar with arts-based methods; early engagement can help support efficacious and timely knowledge translation and exchange opportunities. This is particularly critical with digital stories shared through virtual events where the important role of community co-researchers as story creators and lived experience educators is often overlooked. In online forums, peer researchers presenting their stories can provide invaluable context and local knowledge insights as experience experts. While there are many online and in-person opportunities for virtual presentations of digital stories, sufficient funding is required to ensure that co-researchers can attend and introduce their DSTs and play an active role in co-designing knowledge translation events.

## Discussion

As a participatory visual method, online digital storytelling has many strengths. Similar to other qualitative research reports on virtual workshops [26, 28, 29], our group experienced the online digital storytelling and knowledge translation activities as engaging, convenient and rewarding project experiences. Co-researchers appreciated the flexibility and convenience of online workshop sessions and between session support with script writing and video production. Researchers have observed that virtual qualitative methods supporting remote participation can be more inclusive by eliminating travel time, childcare requirements and providing a more comfortable and familiar setting [25, 27, 35, 36]. When projects are focused on sensitive topics and engage equity-deserving communities, like this initiative, participating from home may also be preferable to revisiting potentially intimidating and/or retraumatizing institutional environments [27, 37]. However, it is also important that online project teams have planned for access to therapeutic support and resources in addition to 1:1 communications between virtual sessions as it can be difficult to read people in virtual environments. Online digital storytelling can engage often excluded patient populations such as persons who are immunocompromised, who experience mobility issues or require personal support workers to participate in in-person venues [14, 21]. Virtual methods can also reduce status differences that may be more visible during in-person gatherings [38]. Workshops conducted virtually can create a more egalitarian power dynamic downplaying qualitative researcher status differences that may be more evident when meeting in-person and reinforced by appearances, behaviours and research tools such as focus group guides and tape recorders [26, 37, 39, 40]. However, these observations of potential strengths of online DST work assume internet connectivity and access to technology. Project teams should have plans to provide technology and good quality internet access when required. DST workshops include technical training, making this method well suited for individuals who may have lower digital literacy [41, 42]. Training on how to use editing software was included as part of the workshop sessions and DST facilitators providing additional one-on-one support in-between sessions to support digital film creation. This flexible but labour intensive approach ensured that co-researchers with various digital literacy levels were able to participate in the technical aspects of the workshop with adequate training and practice.

Based upon our experiences, trained staff and expert facilitators who are experienced and adept at navigating online spaces are critical to the success of digital storytelling as a virtual qualitative research approach. The value of expert DST facilitators, project staff trained in online digital storytelling methods to support individual story work and a collaborative workshop culture has been highlighted in recent research reports [14, 21] and was critical to the success of this project. With this initiative, project staff, having experienced hands-on online DST training, were comfortable supporting individual and small group homework sessions and working alongside community co-researchers and DST facilitators. Feedback from community co-researchers highlighted the importance of carefully considering the size of a digital storytelling group to allow sufficient time to discuss ideas, share narratives and revise DSTs in progress. These observations align with Lobe’s [43] recommendation of considering a smaller group size when working online due to the complexity of reading nonverbal cues and group dynamics. With our online project, a co-researcher group of seven and staff of five worked well for both large and small group work leveraging online breakout rooms.

Recent overviews of virtual qualitative research have posited that connectivity and empathy are more difficult to establish in online spaces [6, 43, 44] and that more time is required to build trust in virtual group settings when compared to meeting in-person [45]. Both the extended timelines and structured online sharing practices of the breast cancer and diversity digital storytelling initiative were DST workshop features that contributed to connectivity and a dynamic research environment. Community co-researchers appreciated the cadence of weekly meetings as an opportunity to get to know one another at a more relaxed pace than during a three-day intensive workshop period. Check-ins and meetings to assist with the completion of DST tasks contributed to a supportive and collaborative project space aimed to encourage co-researchers to share their stories while also having adequate technical support if desired. While the promising practices outlined in this report undoubtedly contributed to the success of this initiative, the passion, creativity and dedication of the co-researchers, qualitative staff and expert facilitators provided a foundation for exceptional team dynamics and project outcomes. The project team learned so much from co-researchers who provided mentorship and local knowledge about their communities and personal health experiences.

For our team, structured listening and constructive feedback practices were also key components for building rapport and trust in an online workshop setting. In particular, story circle principles that support and protect uninterrupted speaking time for co-researchers, facilitated engaged listening and group connectivity. Online story circle methods that involve sequenced protected speaker time, turn-taking and visual feedback strategies provide a supportive environment for online groups and ensure all members have protected time to contribute [46–48]. Project staff and community co-researchers identified DST facilitation strategies, such as turn-taking, physical expression of support and structured feedback strategies, as contributing to connectivity and rapport in online workshop spaces. These DST facilitation strategies resonate with recent research emphasizing the importance of engaged listening practices as supporting more democratized and equity-based virtual research environments [46, 47]. 

The lessons learned throughout this project were generated through debrief conversations and iterative feedback from the project team including co-researchers, qualitative researchers and DST facilitators. Importantly, when adapting digital storytelling to online settings, teaching co-researchers with limited training and prior experience with digital editing does require additional time and budget allocation. Our group benefited from working with professional DST facilitators and filmmakers who were adept with implementing participatory video creation techniques both during workshop and individual coaching sessions. However, this online work did add to the costs associated with the DST production phase and placed constraints on funding available for other project phases. While the co-researchers communicated their interest in sharing their stories more broadly during the interview phase of the larger project, co-researchers were not involved in developing the DST study design for this project. Due to a limited project budget, there were financial constraints to co-researcher compensation available for both the pre-production planning phase, and post-production knowledge translation activities. While the participatory research continuum can range from being minimally participatory to being fully egalitarian, engaging co-researchers more comprehensively in the development of the DST workshops and knowledge translation phases may have led to greater co-researcher inclusivity in all project processes.

## Conclusion

We have reported here on observations and lessons learned from an online DST project conducted during the pandemic with the hope of contributing to a growing knowledge base supporting promising practices for virtual, arts-informed qualitative research. Although online digital storytelling has many advantages as a virtual qualitative method, there are also important cautionary considerations. While it is true that online digital storytelling is convenient and saves costs related to travel time, venue fees, refreshments and other in-person workshop expenditures, virtual DST designs are labour intensive and require project staff who have completed preparatory training and have access to professional mentorship. The complexity of online collaborative group work, script writing and video production necessitates the support of skilled and experienced DST facilitators. The time and resources required for collaborative and carefully planned knowledge translation activities are substantial. Early engagement, planning and co-design with community members and other stakeholders during the project conceptualization and pre-workshop development stage can support impactful knowledge translation activities and social change goals.

## Data Availability

No datasets were generated or analysed during the current study.

## References

[1] Gubrium A, Gubrium E (2021). Narrative complexity in the time of covid-19. Lancet.

[2] Gubrium AC (2020). Participant engagement and ethical digital storytelling: the MOCHA moving Forward Study. Int Q Community Health Educ.

[3] Heron G, Steckley L (2020). Digital storytelling using co-production with vulnerable young people. J Social Work.

[4] Lambert J. Digital storytelling: capturing lives, creating community. Routledge; 2013.

[5] Fiddian-Green A et al. Restor (y) ing health: a conceptual model of the effects of digital storytelling. 2019. *20*(4): p. 502–12. 10.1177/1524839918825130 30736703

[6] Davis H, Waycott J, Schleser M. Digital storytelling: designing, developing and delivering with diverse communities, in managing complexity and creating innovation through design. In: Miettinen S, Sarantou M, editors. Managing complexity and creating innovation through design. Routledge; 2019. pp. 15–24.

[7] Lang M, Laing C, Moules N, Estefan A (2019). Words, camera, music, action: a methodology of digital storytelling in a health care setting. Int J Qualitative Methods.

[8] Moreau KA, Eady K, Sikora L, Horsley T (2018). Digital storytelling in health professions education: a systematic review. BMC Med Educ.

[9] Mojtahedzadeh R, Mohammadi A, Emami AH, Zarei A (2021). How digital storytelling applied in health profession education: a systematized review. J Adv Med Educ Professionalism.

[10] Botfield JR, Newman CE, Lenette C, Albury K, Zwi AB (2018). Using digital storytelling to promote the sexual health and well-being of migrant and refugee young people: a scoping review. Health Educ J.

[11] Briant KJ, Halter A, Marchello N, Escareño M, Thompson B (2016). The power of digital storytelling as a culturally relevant health promotion tool. Health Promot Pract.

[12] Loebach J, Tilleczek K, Chaisson B, Sharp B (2019). Keyboard warriors? Visualising technology and well-being with, for and by indigenous youth through digital stories. Visual Stud.

[13] Akard TF, Duffy M, Hord A, Randall A, Sanders A, Adelstein K, Gilmer MJ (2018). Bereaved mothers’ and fathers’ perceptions of a legacy intervention for parents of infants in the NICU. J Neonatal-perinatal Med.

[14] Rolbiecki AJ, Washington KT, Bitsicas KC, Lero CM, Spinner E, Akard TF. (2021). Digital storytelling for bereaved individuals in a virtual setting. OMEGA-Journal Death Dying, 00302228211051524. 10.1177/00302228211051524 34866480

[15] Atakav E, Jarvis L, Marsden L (2020). Researching British [Muslim] values: vernacular politics, digital storytelling, and participant researchers. Int J Qualitative Methods.

[16] Brekke AJ, Joseph R, Aaftaab NG (2021). I address race because race addresses me: women of color show receipts through digital storytelling. Rev Communication.

[17] Rieger KL, West CH, Kenny A, Chooniedass R, Demczuk L, Mitchell KM, Scott SD. (2018). Digital storytelling as a method in health research: a systematic review protocol. Syst Reviews, *7*(1). 10.1186/s13643-018-0704-y PMC5838876 29506568

[18] Shiri A, Howard D, Farnel S (2022). Indigenous digital storytelling: digital interfaces supporting cultural heritage preservation and access. Int Inform Libr Rev.

[19] Lenette C (2019). Arts-based methods in refugee research.

[20] Cullen O, Jenney A, Shiels L, Greer K, Scott K. (2023). Integrating the voices of Youth with lived experience as co-researchers to Improve Research and Practice approaches to childhood experiences of intimate Partner violence. J Family Violence, 1–15.

[21] Durant KL, Kortes-Miller K (2023). And then COVID hit:(re) flexibility of Digital Storytelling in Qualitative Health Research. Int J Qualitative Methods.

[22] Rieger KL, Bennett M, Martin D, Hack TF, Cook L, Hornan B (2021). Digital storytelling as a patient engagement and research approach with first nations women: how the medicine wheel guided our debwewin journey. Qual Health Res.

[23] Heyen NB, Gardecki J, Eidt-Koch D, Schlangen M, Pauly S, Eickmeier O, Bratan T. (2022). Patient science: Citizen Science involving chronically ill people as co-researchers. J Participatory Res Methods, *3*(1).

[24] Ibáñez-Carrasco F, Watson JR, Tavares J. *Supporting peer researchers: recommendations* Ibáñez-Carrasco, F., Watson, J. R., & Tavares, J. (2019). Supporting peer researchers: recommendations from our lived experience/expertise in community-based research in Canada. *Harm Reduction Journal*, *16*(1), 1–5. 10.1186/s12954-019-0322-6 PMC6724244 31481067

[25] Valdez ES, Gubrium A (2020). Shifting to virtual CBPR protocols in the time of corona virus/COVID-19. Int J Qualitative Methods.

[26] Howlett M (2022). Looking at the ‘field’through a zoom lens: methodological reflections on conducting online research during a global pandemic. Qualitative Res.

[27] LaMarre A, Rice C, Friedman M, Fowlie H (2022). Carrying stories: digital storytelling and the complexities of intimacy, relationality, and home spaces. Qualitative Res Psychol.

[28] Dodds S, Hess AC (2020). Adapting research methodology during COVID-19: lessons for transformative service research. J Service Manage.

[29] Hall J, Gaved M, Sargent J (2021). Participatory research approaches in times of Covid-19: a narrative literature review. Int J Qualitative Methods.

[30] Davey NG, Benjaminsen G (2021). Telling tales: digital storytelling as a tool for qualitative data interpretation and communication. Int J Qualitative Methods.

[31] Sitter KC, Beausoleil N, McGowan E (2020). Digital storytelling and validity criteria. Int J Qualitative Methods.

[32] West, C. H., Rieger, K. L., Kenny, A., Chooniedass, R., Mitchell, K. M., Winther Klippenstein,A., … Scott, S. D. (2022). Digital storytelling as a method in health research: A systematic review. *International Journal of Qualitative Methods*, *21*, 16094069221111118.

[33] Patel A, Regier K, Wilson K, Ghassemi E, Dean J (2018). Beyond the cosmopolis: sustaining hyper-diversity in the suburbs of Peel Region, Ontario. Urban Plann.

[34] Health Experiences Website. Digital storytelling. Retrieved from https://healthexperiences.ca/digital-storytelling/. Accessed 4 Apr 2024.

[35] Pocock T, Smith M, Wiles J (2021). Recommendations for virtual qualitative health research during a pandemic. Qual Health Res.

[36] Sattler, C., Rommel, J., Chen, C., García-Llorente, M., Gutiérrez-Briceño, I., Prager,K., … Kelemen, E. (2022). Participatory research in times of COVID-19 and beyond:Adjusting your methodological toolkits. *One Earth*, *5*(1), 62–73. 10.1016/j.oneear.2021.12.006 PMC8779601 35098107

[37] Keen S, Lomeli-Rodriguez M, Joffe H (2022). From challenge to opportunity: virtual qualitative research during COVID-19 and beyond. Int J Qualitative Methods.

[38] Fox F. Meeting in virtual spaces: Conducting online focus groups. *Collecting qualitative data: a practical guide to textual, media and virtual techniques*. In: Braun V, Clarke V, Gray D, editors. Collecting qualitative data: a practical guide to textual, media and virtual techniques. Cambridge University Press; 2017. pp. 275–99.

[39] Nind M, Coverdale A, Meckin R. (2022). Changing Social Research Practices in the Context of Covid-19: Updated rapid evidence review–synthesis of the 2021 literature. *Updated Rapid Evidence Review–Synthesis of the 2021 Literature* 2022.

[40] Salmons J (2021). Doing qualitative research online.

[41] Lobe B, Morgan D, Hoffman KA (2020). Qualitative data collection in an era of social distancing. Int J Qualitative Methods.

[42] Kulla-Abbott TM. (2007). Developing literacy practices through digital storytelling.

[43] Hausknecht S, Vanchu-Orosco M, Kaufman D. (2017). Sharing life stories: Design and evaluation of a digital storytelling workshop for older adults. In *Computers Supported Education: 8th International Conference, CSEDU 2016, Rome, Italy, April 21–23, 2016, Revised Selected Papers 8* (pp. 497–512). Springer International Publishing.

[44] Meskell P, Houghton C, Biesty L (2021). Opening windows behind closed doors: reflections on working qualitatively during a pandemic. Int J Qualitative Methods.

[45] Rivera A, Okubo Y, Harden R, Wang H, Schlehofer M. (2022). Conducting virtual Youth-Led Participatory Action Research (YPAR) during the COVID-19 pandemic. J Participatory Res Methods, *3*(3, Youth-themed Special Issue).

[46] Motzkau JF, Lee NM (2023). Cultures of listening: psychology, resonance, justice. Rev Gen Psychol.

[47] Ratnam C (2019). Listening to difficult stories: listening as a research methodology. Emot Space Soc.

[48] Żadkowska M, Dowgiałło B, Gajewska M, Herzberg-Kurasz M, Kostecka M. The sociological confessional: a reflexive process in the transformation from face-to-face to online interview. Int J Qualitative Methods. 2022;21:16094069221084785.

